# Macrophage CD40 signaling drives experimental autoimmune encephalomyelitis

**DOI:** 10.1002/path.5205

**Published:** 2019-01-30

**Authors:** Suzanne ABM Aarts, Tom TP Seijkens, Pascal JH Kusters, Claudia M van Tiel, Myrthe E Reiche, Myrthe den Toom, Linda Beckers, Cindy PAA van Roomen, Menno PJ de Winther, Gijs Kooij, Esther Lutgens

**Affiliations:** ^1^ Department of Medical Biochemistry, Subdivision of Experimental Vascular Biology Amsterdam University Medical Centers, Amsterdam Cardiovascular Sciences (ACS), University of Amsterdam Arizona Amsterdam The Netherlands; ^2^ Institute for Cardiovascular Prevention (IPEK), Ludwig Maximilians University (LMU) Munich Germany; ^3^ Department of Molecular Cell Biology and Immunology Amsterdam University Medical Centers, MS Center Amsterdam, Amsterdam Neuroscience Amsterdam The Netherlands

**Keywords:** CD40, TNF receptor‐associated peptides and proteins, macrophages, experimental autoimmune encephalomyelitis, multiple sclerosis

## Abstract

The costimulatory CD40L–CD40 dyad plays a major role in multiple sclerosis (MS). CD40 is highly expressed on MHCII^+^ B cells, dendritic cells and macrophages in human MS lesions. Here we investigated the role of the CD40 downstream signaling intermediates TNF receptor‐associated factor 2 (TRAF2) and TRAF6 in MHCII^+^ cells in experimental autoimmune encephalomyelitis (EAE). Both *MHCII–CD40–Traf2* ^*−/−*^ and *MHCII–CD40–Traf6* ^*−/−*^ mice showed a reduction in clinical signs of EAE and prevented demyelination. However, only *MHCII–CD40–Traf6* ^*−/−*^ mice displayed a decrease in myeloid and lymphoid cell infiltration into the CNS that was accompanied by reduced levels of TNF‐α, IL‐6 and IFN‐γ. As CD40–TRAF6 interactions predominantly occur in macrophages, we subjected *CD40* ^*flfl*^ *LysM* ^*cre*^ mice to EAE. This myeloid‐specific deletion of CD40 resulted in a significant reduction in EAE severity, reduced CNS inflammation and demyelination.

In conclusion, the CD40–TRAF6 signaling pathway in MHCII^+^ cells plays a key role in neuroinflammation and demyelination during EAE. Concomitant with the fact that CD40–TRAF6 interactions are predominant in macrophages, depletion of myeloid CD40 also reduces neuroinflammation. CD40–TRAF6 interactions thus represent a promising therapeutic target for MS. © 2018 The Authors. *The Journal of Pathology* published by John Wiley & Sons Ltd on behalf of Pathological Society of Great Britain and Ireland.

## Introduction

Multiple sclerosis (MS) is a chronic inflammatory disease of the CNS, characterized by a breakdown of the blood–brain barrier, multifocal inflammatory lesions, demyelination, reactive gliosis, axonal degeneration and neurodegeneration 1, 2. MS affects more than two million people worldwide and is one of the most common causes of neurological disability in young adults 2, 3. The exact etiology of MS is unknown, although it is considered to be an autoimmune disease in which lymphocytes programmed to recognize myelin antigen trigger a cascade of events resulting in the formation of acute inflammatory, demyelinating lesions in myelin sheaths in the white matter areas of the CNS 4.

The costimulatory CD40–CD40L dyad has a critical role in driving immune responses and plays a key role in several chronic inflammatory diseases, such as atherosclerosis, obesity, rheumatoid arthritis and MS 5. Both *CD40l* ^*−/−*^ and *CD40* ^*−/−*^ mice are protected against experimental autoimmune encephalomyelitis (EAE) 6, 7. *CD40* ^*−/−*^ mice have reduced T cell priming against myelin oligodendrocyte glycoprotein (MOG) and these T cells produce less IFN‐γ 8. Furthermore, CD40 deficiency inhibits leukocyte infiltration into the CNS 8. CD40 is present in MS on different cell types, including B cells, monocytes, macrophages, endothelial cells, T cells and CNS‐resident cells 9, 10. Activation of these cells by CD40L results in the secretion of proinflammatory cytokines and chemokines 11, 12, and T cell expansion 13, which promotes the ongoing inflammation in the CNS. Antibody‐mediated blockade of CD40L blocks CNS inflammation and disease progression in mice with relapsing EAE 14, 15. Accordingly, CD40 antagonists repressed EAE onset and disease severity in marmoset monkeys 16. Fueled by the success of these experimental studies, clinical trials were initiated. The treatment of MS patients with the anti‐CD40L mAb IDEC‐131 was successful in a pilot study, although the subsequent phase II trial was halted due to the occurrence of a case of severe thromboembolism in a parallel Crohn's disease trial that evaluated IDEC‐131 17. Thus, blockade of CD40L is not a feasible therapeutic strategy, as it can result in thromboembolic side‐effects due to the disruption of αIIbβ3–CD40L interactions in arterial thrombi 18, 19. Moreover, complete inhibition of the CD40–CD40L dyad may result in immunosuppression 20. However, modulation of CD40 downstream signaling pathways, preferably in a cell type‐specific manner, may offer a strategy to reduce the risk for these side‐effects 19, 20. Upon binding of CD40L, CD40 recruits TNF receptor‐associated factors (TRAFs) to exert signaling to downstream pathways 20, 21. The intracellular domain of CD40 contains a distal binding domain for TRAF2/3/5 and a proximal domain for TRAF6 20, 21. Which signaling pathway is activated depends on the cell type and the cellular environment. Here we unravel the role of the CD40–TRAF2 and the CD40–TRAF6 signaling pathways in neuroinflammation by subjecting mice with site‐directed mutagenesis for the TRAF6 or TRAF2/3/5 binding site on the CD40 intracellular tail in MHCII^+^ cells to EAE. We found that both signaling pathways play an important, but distinct, role in EAE disease development, with a prominent role for CD40–TRAF6 signaling in neuroinflammation. As CD40–TRAF6 signaling is especially important in the macrophage 22, 23, we investigated the role of myeloid‐specific CD40 signaling in EAE, using *CD40* ^*flfl*^ *LysM* ^*cre*^ mice and found that macrophage CD40 is indeed an important driver of EAE.

## Materials and methods

### Mice

*CD40* ^*flfl*^ mice were generated by custom design at Ozgene Pty Ltd (Bentley, WA, Australia). A conditional allele was created by inserting loxP sites upstream of exon 2 and downstream of exon 3. Mice carrying the floxed *CD40* gene were crossed with *LysM* ^*cre*^ mice 24 to obtain myeloid‐specific CD40‐deficient mice (*CD40* ^*flfl*^ *LysM* ^*cr*e^ mice). Littermates not containing the LysMcre driver were used as controls (*CD40* ^*flfl*^ or *WT* mice). *CD40* ^*flfl*^ *LysM* ^*cre*^, *CD40* ^*flfl*^, *MHCII–CD40–T2/3/5* ^*−/−*^, *MHCII–CD40–T6* ^*−/−*^ and *CD40* ^*−/−*^ mice were bred and maintained at the animal facility of the Academic Medical Centre (Amsterdam, the Netherlands). C57Bl6 mice were used as a WT control in the TRAF signaling EAE experiment and purchased from Charles River (Lyon, France). All the experimental procedures were approved by the Ethical Committee for Animal Experiments of the Academic Medical Centre, Amsterdam, the Netherlands (AMC).

### EAE induction

To investigate the effects of macrophage CD40 and the CD40–TRAF pathways in EAE, 12‐week‐old female *CD40* ^*flfl*^ *LysM* ^*cre*^, *CD40* ^*flfl*^, *MHCII–CD40–T2/3/5* ^*−/−*^, *MHCII–CD40–T6* ^*−/−*^, *CD40* ^*−/−*^ mice and *WT* mice had *ad libitum* access to food and water and were housed under a 12 h light/dark cycle. On day 0, mice were immunized subcutaneously with 200 µg of a MOG peptide (MOG_35–55_) emulsified in CFA Complete Freund's Adjuvant supplemented with 4 mg/ml *Mycobacterium tuberculosis* H37Ra (Hooke Laboratories, Lawrence, MA, USA). Mice were injected i.p. on days 0 and 1 with 300 (*CD40* ^*flfl*^ *LysM* ^*cre*^ and *CD40* ^*flfl*^ mice, *n* = 12/group) or 100 ng (*MHCII–CD40–T2/3/5* ^*−/−*^, *MHCII–CD40–T6* ^*−/−*^, *CD40* ^*−/−*^ and *WT* mice, *n* = 15/group) pertussis toxin (Hooke Laboratories). A control group was included that received only CFA and pertussis toxin (*n* = 6). Body weight and neurological symptoms were monitored daily. The symptoms were graded using the following scale: 0 = no neurological abnormalities; 0.5 = partial loss of tail tonus; 1 = complete loss of tail tonus; 2 = hind limb paresis; 3 = partial hind limb paralysis; 4 = complete hind limb paralysis; 4.5 = paralysis up to the diaphragm, 5 = death. Scoring of clinical symptoms was performed by an observer who was blinded for the experimental conditions.

### Immunohistochemistry

The cerebellum and spinal cord were collected and fixed in 4% paraformaldehyde and embedded in paraffin. Inflammation was graded on 4 µm thick H&E‐stained sections. Per mouse, between four and six sections of the spinal cord were analyzed. Immunohistochemistry was performed for CD45 (30‐F11, 1:5000; BD, Breda, the Netherlands), mac‐3 (M3/84, 1:30; BD), CD3 (CD3‐12, 1:100; AbD Serotec, Puchheim, Germany) and FoxP3 (1:50, FJK‐16s; eBioscience, San Diego, CA, USA). The spinal cord and cerebellum were stained with luxol fast blue (0.1% LFB; Sigma Aldrich, Saint Louis, MO, USA) for observation of myelin loss. All stained sections were analyzed by an observer who was blinded to the experimental conditions.

### *In vitro* bone marrow‐derived macrophage culture

*CD40* ^*flfl*^ *LysM* ^*cre*^ and *CD40* ^*flfl*^ mouse bone marrow cells were harvested from the femur and tibia and cultured in RPMI‐1640 with 2 mm l‐glutamine, 10% FCS, penicillin (100 U/ml), streptomycin (100 µg/ml) (Gibco, Waltham, MA, USA) and 15% L929‐conditioned medium. On day 8, cells were harvested, seeded at 500 000 cells/ml and laden with 40 µg/ml (DiI‐labeled) myelin for 1.5 h. Myelin uptake was measured using flow cytometry.

### Antibodies and flow cytometry

*CD40* ^*flfl*^ *LysM* ^*cre*^ and *CD40* ^*flfl*^ bone marrow‐derived macrophages (BMDMs) were treated with FGK45 (CD40 agonistic antibody, 30 µg/ml), IFN‐γ (5 ng/ml) and LPS (100 ng/ml). After 6 h, cells were incubated with CD16/CD32 (clone 93, 1:100; eBioscience) primary antibody diluted in FACS buffer (PBS containing 0.5% BSA and 2 mm EDTA) to prevent nonspecific binding of antibodies to the Fc receptors. Cells were then incubated with fluorescently labeled antibodies for CD40 (clone 3/23, 1:200; BioLegend, San Diego, CA, USA), CD80 (16‐10A1, 1:200; BD Pharmingen, Breda, the Netherlands), CD86 (GL‐1, 1:100; BioLegend), MHCII (M5/114.15.2, 1:100; eBioscience), CD36 (MF3, 1:100; Bio‐Rad, Hercules, CA, USA) and CD204 (2F8, 1:100; Bio‐Rad). Blood was obtained by cardiac puncture and collected using EDTA‐filled syringes from *CD40* ^*flfl*^ *LysM* ^*cre*^ and *CD40* ^*flfl*^ mice, *MHCII–CD40–T2/3/5* ^*−/−*^, *MHCII–CD40–T6* ^*−/−*^, *CD40* ^*−/−*^ mice and *WT* mice. Spleen was collected from *MHCII–CD40–T2/3/5* ^*−/−*^, *MHCII–CD40–T6* ^*−/−*^, *CD40* ^*−/−*^ and *WT* mice. Erythrocytes in blood and spleen were removed by incubation with hypotonic lysis buffer (8.4 g NH_4_Cl and 0.84 g NaHCO_3_ per liter of distilled water). Prior to labeling, cell suspensions were incubated with a CD16/32 antibody (clone 93, 1:100; eBioscience). CD45 (30‐F11, 1:200; eBioscience), CD19 (1D3, 1:100; eBioscience), CD3 (145‐2C11, 1:100; BioLegend), CD8 (53–6.7, 1:100; eBioscience), CD4 (GK1.5, 1:100; BD), Ly6G (1A8, 1:200; BD), SiglecF (E50‐2440, 1:100; BD) F4/80 (Cl:A3‐1, 1:100; AbD Serotec) and Ly6C (HK1.4, 1:100; eBioscience) antibodies were incubated with the indicated tissues. Staining was analyzed by flow cytometry (FACSCanto II; BD Biosciences, Breda, the Netherlands) and the FlowJo software version 7.6.5. (Tree Star Inc., Ashland, OR, USA).

### Western blot

*CD40* ^*flfl*^ *LysM* ^*cre*^ and *CD40* ^*flfl*^ BMDMs were cultured as described above, treated for 10 min or 24 h with FGK45 (30 µg/ml), IFN‐γ (5 ng/ml) and LPS (100 ng/ml) and subsequently lysed in RIPA lysis buffer. Protein concentrations were determined using the Pierce BCA Protein Assay Kit (Thermo Scientific, Waltham, MA, USA). Equal amounts of protein samples were loaded on to a SDS polyacrylamide gel and transferred to a nitrocellulose membrane (Bio‐Rad). After blocking with 5% BSA in PBS containing 0.05% Tween‐20, blots were incubated overnight with anti‐CD40 (Abcam, Cambridge, UK) and anti‐α‐tubulin (Cedarlane, Burlington, ON, Canada). Blots were then washed and incubated with the appropriate HRP‐conjugated secondary antibody (1:800; Thermo Scientific) and visualized using the SuperSignal West Pico Plus Chemiluminescent Substrate kit (Thermo Scientific).

### Analysis of Igs

MOG IgG was measured in plasma samples using an anti‐MOG(35–55) IgG Quantitative ELISA Kit (SensoLyte®, Fremont, CA, USA).

### Analysis of cytokine profiles

The production of pro‐ and anti‐inflammatory mediators was assessed by ELISA in cell‐free supernatants of 24 h myelin (40 µg/ml), FGK45 (30 µg/ml), IFN‐γ (5 ng/ml) and LPS (100 ng/ml) or IL‐4 (10 ng/ml) treated *CD40* ^*flfl*^ *LysM* ^*cre*^ and *CD40* ^*flfl*^ BMDMs using commercial kits for human IL‐12, IL‐6 and the TNF‐α CytoSet ELISA Kit (Biosource, Nivelles, Belgium) according to the manufacturers' protocols. The samples were measured using a Luminex 200 (Bio‐Rad).

### RNA isolation and gene expression analysis

Total RNA from the CNS was extracted using TRIzol (Invitrogen, Carlsbad, CA, USA) and RNA from BMDMs was isolated using High Pure RNA Isolation Kits (Roche, Basel, Switzerland). RNA was reverse transcribed using an iScript cDNA synthesis kit (Bio‐Rad) and quantitative (q)PCR was performed using a SYBR Green PCR Kit (Applied Biosystems, Leusden, the Netherlands) on a ViiA7 real‐time PCR system (Applied Biosystems). Expression levels of transcripts obtained using RT‐qPCR were normalized to the mean expression levels of the reference transcripts *Gapdh* and *Rplp0*. Primer sequences can be found in supplementary material, Table S1.

### Statistical analysis

Results are presented as mean ± SEM. Calculations were performed using GraphPad Prism 7.0 software (GraphPad Software Inc., San Diego, CA, USA). Statistical significance was evaluated using unpaired Student's *t*‐tests. Clinical parameters were analyzed by nonparametric (Mann–Whitney) test. *P* values < 0.05 were considered statistically significant. *n* represents the number of biological replicates.

## Results and discussion

### Both CD40–TRAF6 and CD40–TRAF2 signaling pathways drive EAE

**Figure 1:**
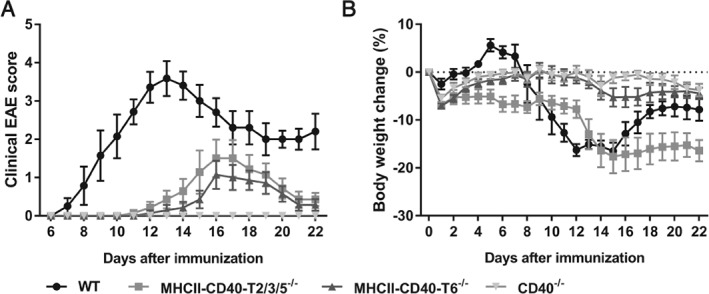
Deficiency of CD40, the CD40–TRAF2/3/5 interaction and the CD40–TRAF6 interaction is protective in EAE. (A) Clinical EAE scores of *MHCII–CD40–T2/3/5* ^*−/−*^ (*n* = 7), *MHCII–CD40–T6* ^*−/−*^ (*n* = 7), *CD40* ^*−/−*^ (*n* = 7) and *WT* (*n* = 7) mice 22 days after EAE induction. (B) Percentage body weight change compared with day 0. Mean ± SEM.

To define which CD40 signaling pathways are involved in neuroinflammation, we investigated the effects of disruption of either CD40–TRAF6 or CD40–TRAF2 signaling in antigen‐presenting cells in EAE. CD40‐deficient mice expressing a chimeric CD40 transgene with mutations at the TRAF6 or TRAF2/3/5 binding site under the control of the MHCII promotor (*MHCII–CD40–T6* ^*−/−*^, *MHCII–CD40–T2/3/5* ^*−/−*^) 25, CD40‐deficient mice (*CD40* ^*−/−*^) and C57Bl6 WT mice (*WT*) were subjected to EAE. In accordance with previous studies 8, *CD40* ^*−/−*^ mice were completely protected against EAE (Figure 1A and Table 1). Compared with *WT* mice, disease severity was significantly lower in both the *MHCII–CD40–T2/3/5* ^*−/−*^ and *MHCII–CD40–T6* ^*−/−*^ mice and was found to be lowest in the *MHCII–CD40–T6* ^*−/−*^ mice (maximum disease score 4.2 ± 0.2 in *WT* versus 2.7 ± 0.4 in *MHCII–CD40–T2/3/5* ^*−/−*^ and 1.8 ± 0.3 in *MHCII–CD40–T6* ^*−/−*^, *p* = 0.0049; Figure 1A and Table 1). Consistently, the cumulative disease score (area under the curve; AUC) of the *MHCII–CD40–T2/3/5* ^*−/−*^, *MHCII–CD40–T6* ^*−/−*^ and *CD40* ^*−/−*^ mice was significantly lower than the cumulative score of the *WT* mice (Table 1). Moreover, the average disease onset was >4 days delayed in the *MHCII–CD40–T2/3/5* ^*−/−*^ and *MHCII–CD40–T6* ^*−/−*^ mice compared with *WT* mice (onset on day 13.4 ± 0.9 and day 14.6 ± 0.7 versus day 8.9 ± 0.6, *p* < 0.05) (Figure 1A and Table 1). The incidence of EAE in *WT* mice was 100.0, 71.4% in both *MHCII–CD40–T2/3/5* ^*−/−*^ and *MHCII–CD40–T6* ^*−/−*^ mice and 0% in *CD40* ^*−/−*^ mice (*p* < 0.05, Table 1). Survival rates were only 71.4% for *WT* mice, whereas all *MHCII–CD40–T2/3/5* ^*−/−*^, *MHCII–CD40–T6* ^*−/−*^ and *CD40* ^*−/−*^ mice survived until the end of the experiment (Table 1). Additionally, in the *MHCII–CD40–T6* ^*−/−*^ mice we observed only 5.3 ± 1.9% body weight loss (*p* < 0.001, Figure 1B and Table 1) at the peak of disease compared with day 0, whereas *WT* mice suffered an average loss of 16.9 ± 1.4% (Figure 1B and Table 1). Comparable with *WT* mice, the *MHCII–CD40–T2/3/5* ^*−/−*^ suffered an average of 17.6 ± 3.5% body weight loss (Figure 1B and Table 1). *CD40* ^*−/−*^ mice only had 1.2 ± 1.1% body weight loss (Figure 1B and Table 1). *WT* mice displayed extensive CNS demyelination compared with *CD40* ^*−/−*^, *MHCII–CD40–T6* ^*−/−*^ and *MHCII–CD40–T2/3/5* ^*−/−*^ mice, as shown by LFB staining (see supplementary material, Figure S1A). These data confirm that CD40 plays a pivotal role in EAE development and reveal that both CD40–TRAF2/3/5 the CD40–TRAF6 signaling pathways are involved in its pathogenesis.

**Table 1:** EAE disease measurements of *MHCII–CD40–T2/3/5* ^*−/−*^, *MHCII–CD40–T6* ^*−/−*^, *CD40* ^*−/−*^ mice and *WT* mice

Group	Incidence (%)	Survival (%)	AUC	Mean day of disease onset	Mean maximum disease score	Body weight loss (%)
WT	100.0	71.4	30.8	8.9	4.2	16.9
MHCII–CD40–T2/3/5^−/−^	71.4	100.0	8.0**	13.4**	2.7*	17.6
MHCII–CD40–T6^−/−^	71.4	100.0	5.0***	14.6**	1.8**	5.3***
CD40^−/−^	0.0	100.0	0.0***	N/A	N/A	1.2***

*p < 0.05, **p < 0.01 as determined by the nonparametric Mann–Whitney *U*‐test for disease scores. Percentage body weight loss was calculated from day 15 compared with day 0 and significance was determined by Student's *t*‐test; ***p < 0.001 versus WT.

### CD40‐, CD40–TRAF6‐ and CD40–TRAF2/3/5‐deficient mice have impaired anti‐MOG IgG production

**Figure 2:**
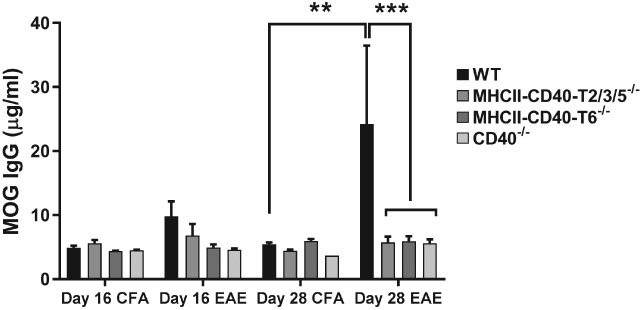
CD40‐, CD40–TRAF6‐ and CD40–TRAF2/3/5‐deficient mice have impaired anti‐MOG IgG production. MOG‐specific IgG concentrations in serum of *MHCII–CD40–T2/3/5* ^*−/−*^, *MHCII–CD40–T6* ^*−/−*^, *CD40* ^*−/−*^ and *WT* mice 16 days after induction (*n =* 8/group) and 28 days after induction (*n =* 7/group). Mean ± SEM. *p < 0.05, **p < 0.01, ***p < 0.001 versus WT.

As CD40 signaling plays an important role in isotype switching 26 we analyzed MOG‐specific IgG levels in the plasma of mice immunized for EAE. Twenty‐eight days after EAE induction, MOG‐specific IgG titers were significantly lower in mice lacking CD40, CD40–T2/3/5 or CD40–T6 signaling compared with *WT* mice (Figure 2), confirming the impaired Ig isotype switching in the absence of CD40, CD40–TRAF2/3/5 or CD40–TRAF6 signaling.

### CD40‐ and CD40–TRAF6‐deficient mice are protected against neuroinflammation

**Figure 3:**
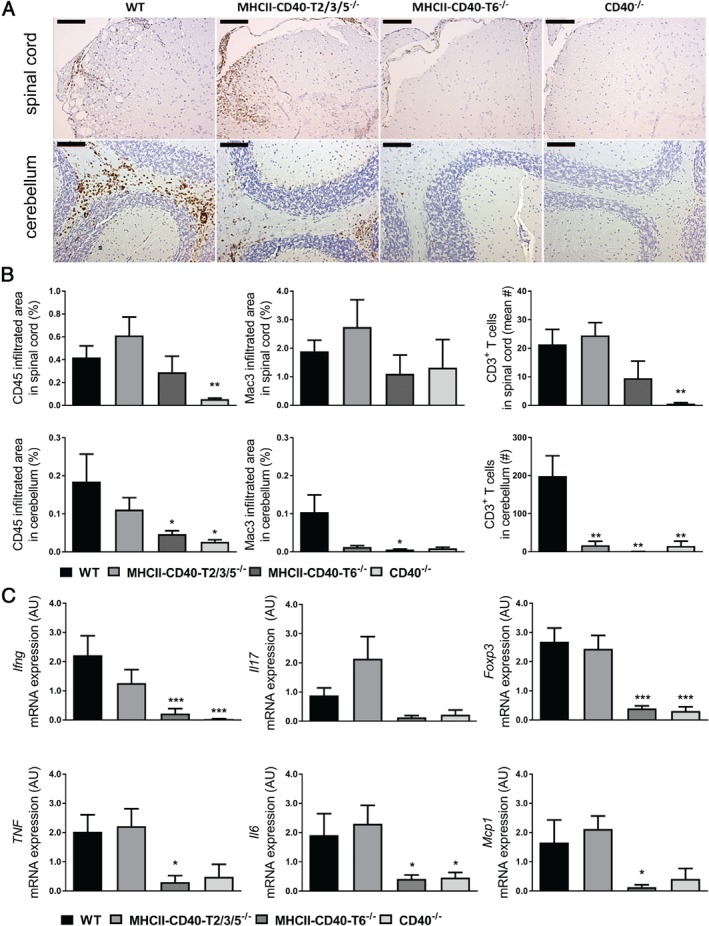
CD40‐ and CD40–TRAF6‐deficient mice exhibit reduced neuroinflammation. (A) Representative images of CD45^+^ staining of spinal cord and cerebellum sections, bars 100 µm. (B) Quantification of CD45^+^, mac‐3^+^ and CD3^+^ immune cell infiltrates in the spinal cord and cerebellum from mice sacrificed at day 16 (*n =* 8/group). (C) mRNA expression levels of inflammatory genes in cerebellum obtained 16 days after induction of EAE, presented as relative expression compared with *Gapdh* and *Rplp0*. *CD40* ^*−/−*^ and *MHCII–CD40–T6* ^*−/−*^ mice, but not *MHCII–CD40–T2/3/5* ^*−/−*^ mice, had significantly reduced inflammatory cytokine expression in the CNS during EAE compared with *WT* mice (*n =* 8/group). Mean ± SEM. *p < 0.05, **p < 0.01, ***p < 0.001 versus WT.

Analysis of the CNS (cerebellum and spinal cord) shows that the number of total leukocytes (CD45^+^ cells), macrophages (Mac3^+^ cells) and T cells (CD3^+^ cells) that infiltrated the CNS was significantly reduced in *MHCII–CD40–T6* ^*−/−*^ and *CD40* ^*−/−*^ mice compared with *WT* mice subjected to EAE (Figure 3A,B). Although *MHCII–CD40–T2/3/5* ^*−/−*^ mice exhibited significantly milder EAE disease symptoms than *WT* mice, the amount of immune cell infiltration in the CNS did not differ (Figure 3A,B). Moreover, although not significant, the number of CD45^+^ cells in the spinal cord of *WT* versus *MHCII–CD40–TRAF2/3/5* ^*−/−*^ mice even seemed to be increased (Figure 3A,B), whereas clinical symptoms were ameliorated. One of the possible reasons why this increase in CD45^+^ cells did not result in an aggravated EAE response is that the number of immune‐regulatory FoxP3^+^ T cells was also increased (see supplementary material, Figure S1B), resulting in a ‘balanced’ immune response 27. In a previous study, in which *MHCII–CD40–T2/3/5* ^*−/−*^ mice were subjected to atherosclerosis, we also observed an increase in CD3^+^ T cells during disease and found that the increase was due to an increase in regulatory T cells 22. The balancing of the immune response is also reflected by the fact that gene expression analysis also showed no differences in the expression of inflammatory genes in the CNS of these mice (Figure 3C). The reduction in EAE severity in the *MHCII–CD40–T2/3/5* ^*−/−*^ mice might be explained by the reduced MOG IgG production by the B cells (Figure 2).

Both *CD40* ^*−/−*^ and *MHCII–CD40–T6* ^*−/−*^ mice had significantly reduced *Ifng*, *Foxp3*, *Tnf*, *Il6* and *Mcp1* expression in the CNS compared with *WT* or *MHCII–CD40–T2/3/5* ^*−/−*^ mice during the peak of disease (Figure 3C). *MHCII–CD40–T2/3/5* ^*−/−*^ mice did not show any changes in CNS cytokine levels (Figure 3C). Analysis of the peripheral immune response in blood and spleen showed only minor changes (see supplementary material, Figure S2). In *MHCII–CD40–T6* ^*−/−*^ mice, we observed an increase in the percentage of CD19^+^ (B) cells in blood and spleen (see supplementary material, Figure S2A,B) and a decreased percentage of Ly6G^+^ cells (neutrophils) in blood compared with *WT* mice during EAE. This decrease in Ly6G^+^ cells (neutrophils) was also observed in *MHCII–CD40–T2/3/5* ^*−/−*^ mice.

Taken together, our data reveal that both CD40–TRAF6 and CD40–TRAF2/3/5 signaling pathways in MHCII^+^ cells are involved in the pathogenesis of EAE. Although both *MHCII–CD40–T2/3/5* ^*−/−*^ and *MHCII–CD40–T6* ^*−/−*^ mice exhibited a lower disease score, only *MHCII–CD40–T6* ^*−/−*^ mice were protected against EAE‐associated body weight loss and CNS inflammation, indicating that CD40–TRAF6 signaling is more relevant in EAE than CD40–TRAF2/3/5 signaling.

The CD40–TRAF6 pathway is especially known to be important for CD40 signaling in the myeloid lineage. CD40–TRAF6 interactions induce classical monocyte activation, monocyte recruitment and macrophage activation via canonical NF‐κB signaling 23, 27, 28, 29. Although the role of CD40–CD40L signaling in MS is mostly attributed to T cells 30 and B cells 31, the main cell types expressing CD40 in human MS lesions are monocytes, macrophages and activated microglia 7. Infiltrating macrophages and activated microglia present in active human MS lesions contribute to CNS injury and have mainly M1 characteristics, including high CD40 expression 32, 33, 34. Although macrophage CD40 is abundantly present in MS lesions, its role in the pathogenesis of MS is currently unknown.

### Myeloid CD40‐deficient mice develop less severe EAE and exhibit decreased CNS inflammation

**Figure 4:**
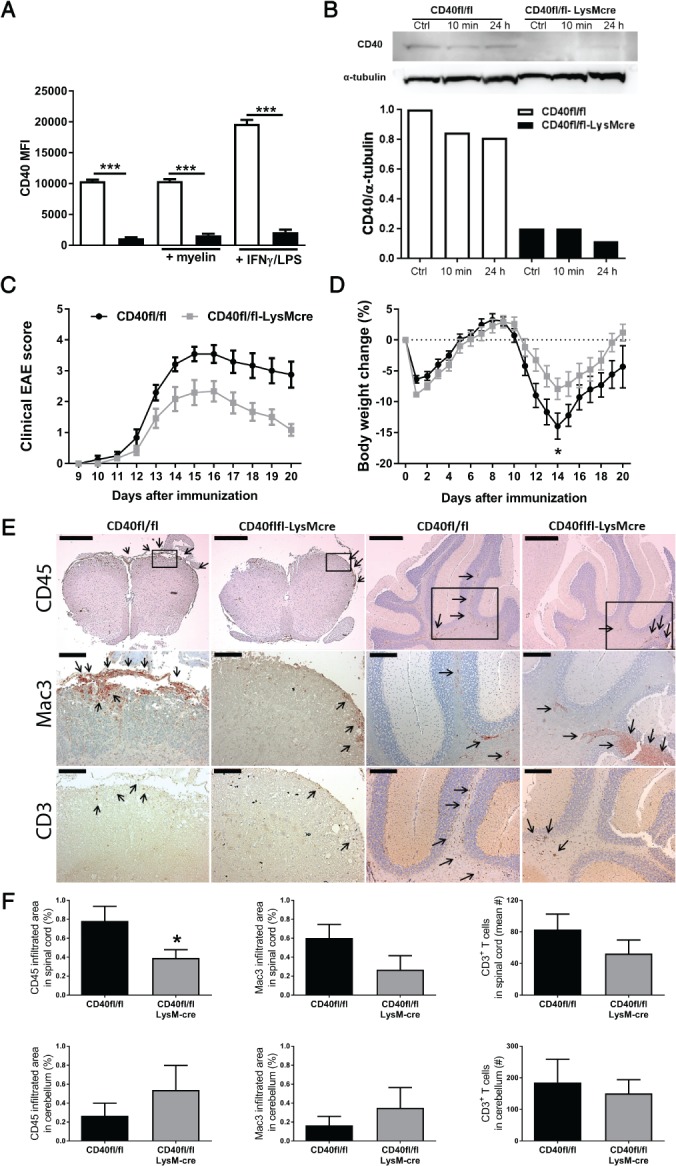
Myeloid CD40 deficiency ameliorates EAE. BMDMs from *CD40* ^*flfl*^ *LysM* ^*cre*^ mice and *CD40* ^*flfl*^ (WT) mice were stimulated for 6 h with FGK45 (30 µg/ml) and myelin (40 µg/ml) or IFN‐γ (5 ng/ml) and LPS (100 ng/ml). (A) MFI of CD40 measured by flow cytometry. (B) Western blot of CD40 (40 kDa band size) and α‐tubulin (50 kDa band size) and quantification of CD40 protein expression in *CD40* ^*flfl*^ *LysM* ^*cre*^ mice and *CD40* ^*flfl*^ (WT) BMDMs stimulated with FGK45 for 10 min or 24 h. (C) Clinical EAE scores of *CD40* ^*flfl*^ *LysM* ^*cre*^ mice and *CD40* ^*flfl*^ (WT) mice during the 20 days after induction (*n* = 12/group). (D) Percentage body weight change compared with the start weight at day 0. (E) Representative images of CD45, MAC3 and CD3 immunostaining of spinal cord and cerebellum sections. CD45: bars, spinal cord and cerebellum, 500 µm. MAC3 and CD3: bars, spinal cord, 100 µm; cerebellum, 200 µm. (F) Counts of CD45 (leukocytes), mac‐3 (macrophages) and CD3 (T cells) immune cell infiltrates in the spinal cord and cerebellum. Means ± SEM. The *in vitro* results in (A) and (B) are means ± SEM from experimental triplicates and are representative of at least three independent experiments. *p < 0.05 versus WT.

To study the role of myeloid CD40 on neuroinflammation *in vivo*, *CD40* ^*flfl*^ *LysM* ^*cre*^ mice were generated, which exhibited an 84–89% reduction in CD40 expression in macrophages (Figure 4A,B). We immunized *CD40* ^*flfl*^ *LysM* ^*cre*^ and *CD40* ^*flfl*^ (*WT*) mice for induction of EAE. Deficiency of myeloid CD40 resulted in significant lower disease severity (maximum disease score 3.0 ± 0.2) and cumulative disease score (AUC 14.4 ± 2.0) compared with *WT* controls (maximum score 4.0 ± 0.3, *p* = 0.010, AUC 24.7 ± 1.9, *p* = 0.0013; Figure 4C and Table 2). Moreover, survival of *CD40* ^*flfl*^ *LysM* ^*cre*^ mice was increased compared with *WT* mice (100% versus 75%, Table 2). In *WT* mice, the maximum body weight loss was 13.9 ± 2.0% and resulted in a 4.3 ± 3.4% weight loss at the end of the experiment. *CD40* ^*flf*^ *LysM* ^*cre*^ mice had significantly less severe weight loss, with a maximum of only 7.9 ± 1.7% loss and complete recovery of body weight at the end of the experiment (*p* < 0.05, Figure 4D and Table 2). As expected from a macrophage‐specific CD40 knockout, IgM, total IgG and anti‐MOG IgG levels did not differ between *CD40* ^*flfl*^ *LysM* ^*cre*^ and *WT* mice (see supplementary material, Figure S3). The CNS of *CD40* ^*flfl*^ *LysM* ^*cre*^ mice exhibited a significant reduction in CD45^+^ leukocyte infiltration, which was due to a reduction in Mac3^+^ macrophages/active microglia and CD3^+^ T cells (Figure 4E,F), revealing a reduction in CNS inflammation in the absence of macrophage CD40. No differences in peripheral immune responses were observed (see supplementary material, Figure S4C). In addition to the previously described role of CNS CD40 on microglial cell activation during EAE 35, we here show that loss of peripheral macrophage CD40 signaling ameliorates EAE and prevents inflammation, revealing a key role for macrophage CD40 signaling in neuroinflammation.

**Table 2:** EAE disease measurements of *CD40* ^*flfl*^ *LysM* ^*cre*^ mice and *WT* mice

Group	Incidence (%)	Survival (%)	AUC	Mean day of disease onset	Mean maximum disease score	Body weight loss (%)
WT	85.7	75.0	24.7	12.2	4.0	13.9
CD40^flfl^LysMcre	80.0	100.0	14.4**	12.8	3.0*	7.9*

Percentage body weight loss was calculated from day 14 compared with day 0. *p < 0.05, **p < 0.01 versus WT as determined by the nonparametric Mann–Whitney *U*‐test for disease scores or Student's *t*‐test for body weight loss.

**Figure 5:**
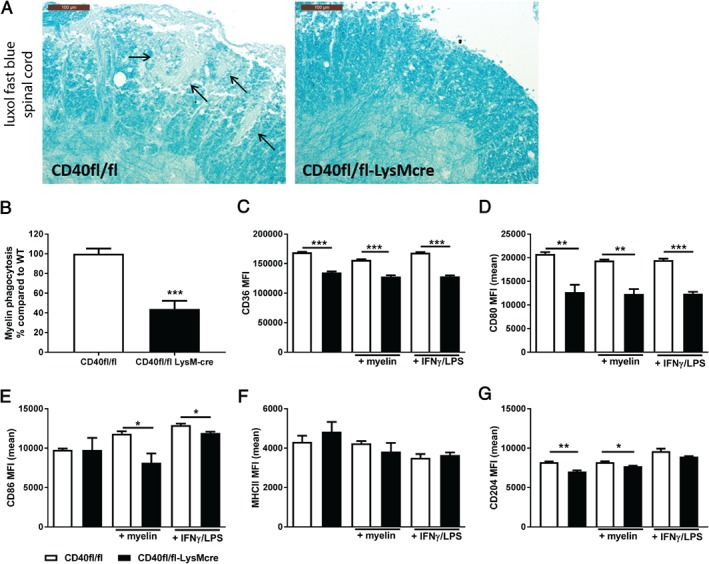
CD40‐deficient macrophages have decreased myelin phagocytosis capacity and display reduced costimulatory capacity. (A) Representative LFB staining of spinal cord; arrows indicate areas with loss of myelin; bars 100 µm. (B) Myelin phagocytosis capacity of *CD40* ^*flfl*^ *LysM* ^*cre*^ mice and *CD40* ^*flfl*^ (*WT*) BMDMs. BMDMs from *CD40* ^*flfl*^ *LysM* ^*cre*^ mice and *WT* mice were stimulated for 6 h with FGK45 (30 µg/ml) and myelin (40 µg/ml) or IFN‐γ (5 ng/ml) and LPS (100 ng/ml). Flow cytometry analysis of (C) CD36, (D) CD80, (E) CD86, (F) MHCII, and (G) CD204 expression of *CD40* ^*flfl*^ *LysM* ^*cre*^ mice and *WT* BMDMs. *In vitro* data in (B)–(F) are means ± SEM from experimental triplicates and are representative of at least two independent experiments. *p < 0.05, **p < 0.01, ***p < 0.001 versus *WT*.

In EAE, myeloid cells cause CNS damage by presenting antigen, secretion of cytokines and phagocytosis of cell debris, resulting in the production of free radicals 36. In EAE lesions, macrophages phagocytose myelin and allow the removal of cell debris, critical for effective repair 33. CNS LFB staining displayed that *CD40* ^*flfl*^ *LysM* ^*cr*e^ mice were protected against EAE‐induced myelin loss (Figure 5A). Flow cytometry analysis of myelin‐laden BMDMs from *CD40* ^*flfl*^ *LysM* ^*cre*^ and *WT* mice show that *CD40* ^*flfl*^ *LysM* ^*cre*^ BMDMs ingested significantly less myelin than *WT* BMDMs (54.8 *±* 8.6% reduction, *n* = 4) (Figure 5B). *CD40* ^*flfl*^ *LysM* ^*cre*^ BMDMs also expressed less CD36, suggesting that macrophage CD40 mediates clearance of myelin debris (Figure 5C). Earlier research using bone marrow chimeric mice showed that CD40 expression on hematopoietic cells is essential for the priming of encephalitogenic T cells 8. FACS analysis revealed that *CD40* ^*flfl*^ *LysM* ^*cre*^ BMDMs exhibited a reduced expression of the classically activated macrophage markers CD80 and CD86, an unchanged expression of MHCII and a decrease in the scavenger receptor and, alternatively, the activated macrophage marker CD204 (Figure 5D–G). The reduced expression of costimulatory molecules supports the results on the reduced EAE development in these mice as costimulation is an essential signal for T cell activation 37, 38. Gene expression analysis of BMDMs incubated with myelin showed no significant differences (see supplementary material, Figure S4A,B). mRNA levels of *Il12b* were decreased in *CD40* ^*flfl*^ *LysM* ^*cre*^ macrophages treated with LPS/IFN‐γ, whereas *Il6*, *Tnf* and *Nos2* mRNA levels were increased (see supplementary material, Figure S4A). However, protein levels of these cytokines were not affected (see supplementary material, Figure S4C). *CD40* ^*flfl*^ *LysM* ^*cre*^ macrophages treated with IL‐4 showed increased *Cdh1* gene expression compared with *WT* BMDMs, but no differences were observed in mRNA levels of other immunomodulatory genes, including *Cd204*, *Il10* and *Tgfb* (see supplementary material, Figure S4B). In MS lesions, human foamy macrophages and microglia express proinflammatory (CD40 and iNOS) markers as well as anti‐inflammatory markers (MR) 33. Macrophages are diverse and plastic, their phenotype is dependent on cues from the microenvironment, including inflammatory signals 39. This might explain why we do not observe a reduced inflammatory phenotype *in vitro*, whereas the *CD40* ^*flfl*^ *LysM* ^*cre*^ mice display reduced neuroinflammation and are protected against clinical symptoms of EAE *in vivo*.

## Conclusions

We have shown that macrophage CD40 plays a key role during neuroinflammation, probably via CD40–TRAF6 signaling. However, the reduction in neuroinflammation was stronger in mice lacking CD40–TRAF6 signaling in all MHCII^+^ cells than in mice lacking CD40 signaling in macrophages, suggesting that both CD40–TRAF6 signaling in macrophages and other antigen‐presenting cells including B cells and dendritic cells drive neuroinflammation. Recently, we have developed small molecule inhibitors of the CD40–TRAF6 interaction, TRAF‐STOPs, and we were able to show that these small molecule inhibitors can reduce atherosclerosis, peritonitis, sepsis and metabolic complications of diet‐induced obesity in mice 23, 28, 29. Interestingly, TRAF‐STOP treatment was also able to reduce CNS leukocyte infiltration during EAE in both mice and rats but unfortunately failed to ameliorate disease severity 40. Further optimization of TRAF‐STOPs for more optimal delivery in the CNS may be required to achieve better therapeutic results.

Our data reveal that blocking CD40 in a cell type‐specific manner, or inhibition of parts of the CD40 signaling, has great therapeutic potential for the treatment of MS, as it will cause limited immune‐suppressive side‐effects compared with untargeted inhibition of the entire CD40 pathway.

## Author contributions statement

The study presented here was carried out in collaboration among all authors. All authors read and approved the final manuscript. SA and TS contributed to the study concept and design, performing experiments, acquisition of data, analysis and interpretation of data, drafting of the manuscript and statistical analysis. PK, CT and MR performed experiments and acquisition of data. MT, LB and CR carried out the acquisition and immunohistochemistry of the CNS. MW, GK and EL contributed to the study concept and design, analysis and interpretation of data, drafting of the manuscript and critical revision of the manuscript for important intellectual content, obtained funding and offered study supervision.

**SUPPLEMENTARY MATERIAL ONLINE**

**Supplementary figure legends**

**Figure S1.** CNS inflammation of MHCII–CD40–T2/3/5^−/−^, MHCII–CD40–T6^−/−^, CD40^−/−^ and WT mice

**Figure S2.** Peripheral immune responses in *MHCII–CD40–T2/3/5* ^*−/−*^, *MHCII–CD40–T6* ^*−/−*^, *CD40* ^*−/−*^, *CD40* ^*flfl*^ *LysM* ^*cre*^ and *WT* mice

**Figure S3.** Normal Ig isotype switching in *CD40* ^*flfl*^ *LysM* ^*cre*^ *mice*

**Figure S4.** CD40‐deficient macrophages have a diverse inflammatory pattern

**Table S1.** Primer sequences

## Supporting information


**Supplementary figure legends**

**Figure S1.** CNS inflammation of *MHCII–CD40–T2/3/5* ^*−/−*^, *MHCII–CD40–T6* ^*−/−*^, *CD40* ^*−/−*^ and *WT* mice

**Figure S2.** Peripheral immune responses in *MHCII–CD40–T2/3/5* ^*−/−*^, *MHCII–CD40–T6* ^*−/−*^, *CD40* ^*−/−*^, *CD40* ^*flfl*^ *LysM* ^*cre*^ and *WT* mice

**Figure S3.** Normal Ig isotype switching in *CD40* ^*flfl*^ *LysM* ^*cre*^ *mice*

**Figure S4.** CD40‐deficient macrophages have a diverse inflammatory pattern

**Table S1.** Primer sequences
