# Supplementary material for: Macrophage CD40 signaling drives experimental autoimmune encephalomyelitis
Source: J Pathol. 2019 Jan 30;247(4):471–80. doi: 10.1002/path.5205 (PMC6519352; doi:10.1002/path.5205)
Supplement: Supplementary file 1 — Supplementary figure legends [file PATH-247-471-s005.docx]

**Macrophage CD40 signaling drives experimental autoimmune encephalomyelitis**

Aarts SABM *et al*. *J Pathol* 2018 (DOI: 10.1002/path.5205)

**Figure S1. CNS inflammation of *MHCII–CD40–T2/3/5^–/–^*, *MHCII–CD40–T6^–/–^*, *CD40^–/–^* and *WT* mice.** (A) Representative images of LFB, CD45, mac-3 and CD3 from spinal cord and cerebellum of *MHCII–CD40–T2/3/5^–/–^*, *MHCII–CD40–T6^−/−^*, *CD40^–/–^* and *WT* mice. Scale bars: spinal cord 200 µm; cerebellum 100 µm. (B) FoxP3 staining of spinal cord and its quantification; scale bars 100 µm. Means ± SEM.

**Figure S2.** **Peripheral immune responses in *MHCII–CD40–T2/3/5^–/–^*, *MHCII–CD40–T6^–/–^*, *CD40^–/–^*, *CD40^flfl^LysM^cre^* and *WT* mice.** Leukocyte subsets of *MHCII–CD40–T2/3/5^–/–^*, *MHCII–CD40–T6^–/–^*, *CD40^–/–^* and *WT* mice in (A) blood and (B) spleen. (C) No difference in blood leukocyte subsets of *CD40^flfl^LysM^cre^* and *WT mice.* Means ± SEM.

**Figure S3. Normal Ig isotype switching in *CD40^flfl^LysM^cre^ mice.*** Total IgM, IgG and MOG-specific IgG concentrations in serum of *CD40^flfl^LysM^cre^* and *WT* mice 28 days after EAE induction. Means ± SEM (*n* = 3/CFA group; *n* = 7–9/EAE group).

**Figure S4. CD40-deficient macrophages have a diverse inflammatory pattern.** *CD40^flfl^LysM^cre^* and *WT* BMDMs were stimulated for 24 h with myelin (M40, 40 µg/ml) or with FGK45, IFNγ (5 ng/ml) and LPS (100 ng/ml). (A) mRNA levels of *Il6*, *Il12b*, *Tnf*, *Nos2* and *Il10*. (B) mRNA levels of *Cd204*, *Cdh1*, *Il10*, and *Tgfb.* (C) Cytokine levels of IL6, IL12 and TNF-α measured in the supernatants after 24 h of IFNγ/LPS stimulation. Means ± SEM. **p* < 0.05 versus *WT*.
