# Supplementary figures and images for: Macrophage CD40 signaling drives experimental autoimmune encephalomyelitis
Source: J Pathol. 2019 Jan 30;247(4):471–80. doi: 10.1002/path.5205 (PMC6519352; doi:10.1002/path.5205)

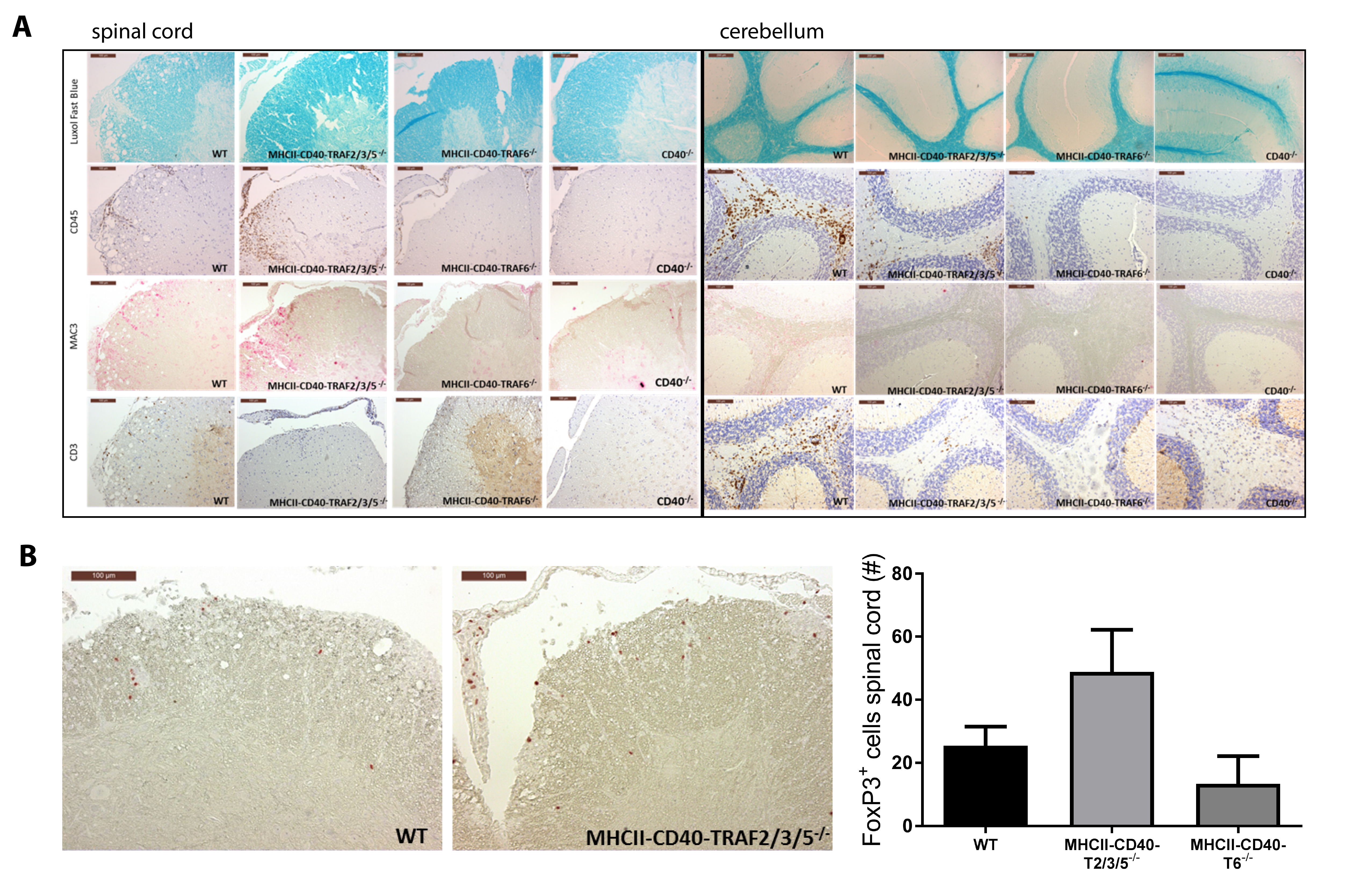

Supplement: Supplementary file 2 — Figure S1. CNS inflammation of MHCII–CD40–T2/3/5 −/−, MHCII–CD40–T6 −/−, CD40 −/− and WT mice [file PATH-247-471-s004.tif]

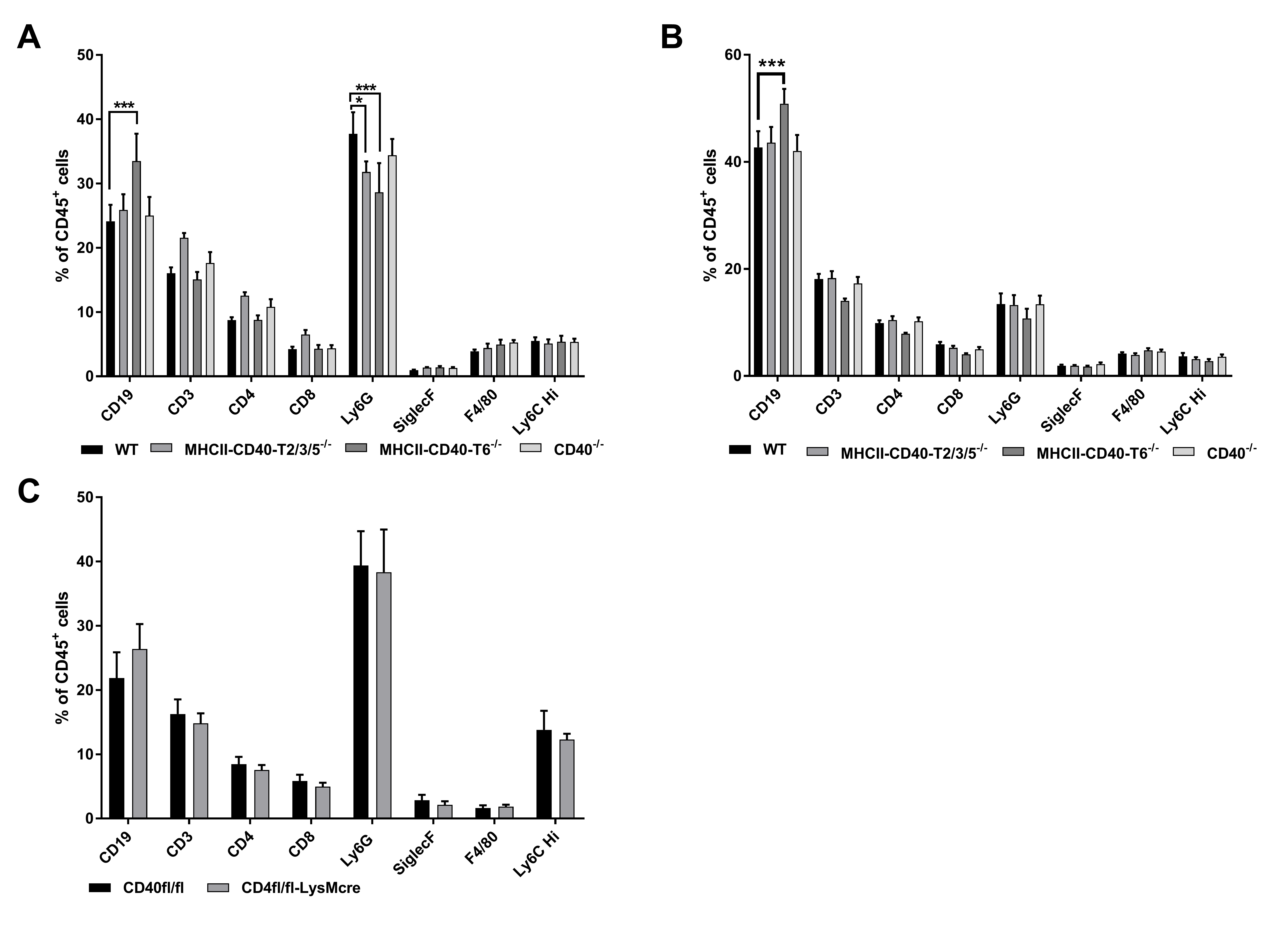

Supplement: Supplementary file 3 — Figure S2. Peripheral immune responses in MHCII–CD40–T2/3/5 −/−, MHCII–CD40–T6 −/−, CD40 −/−, CD40 flfl LysM cre and WT mice [file PATH-247-471-s003.tif]

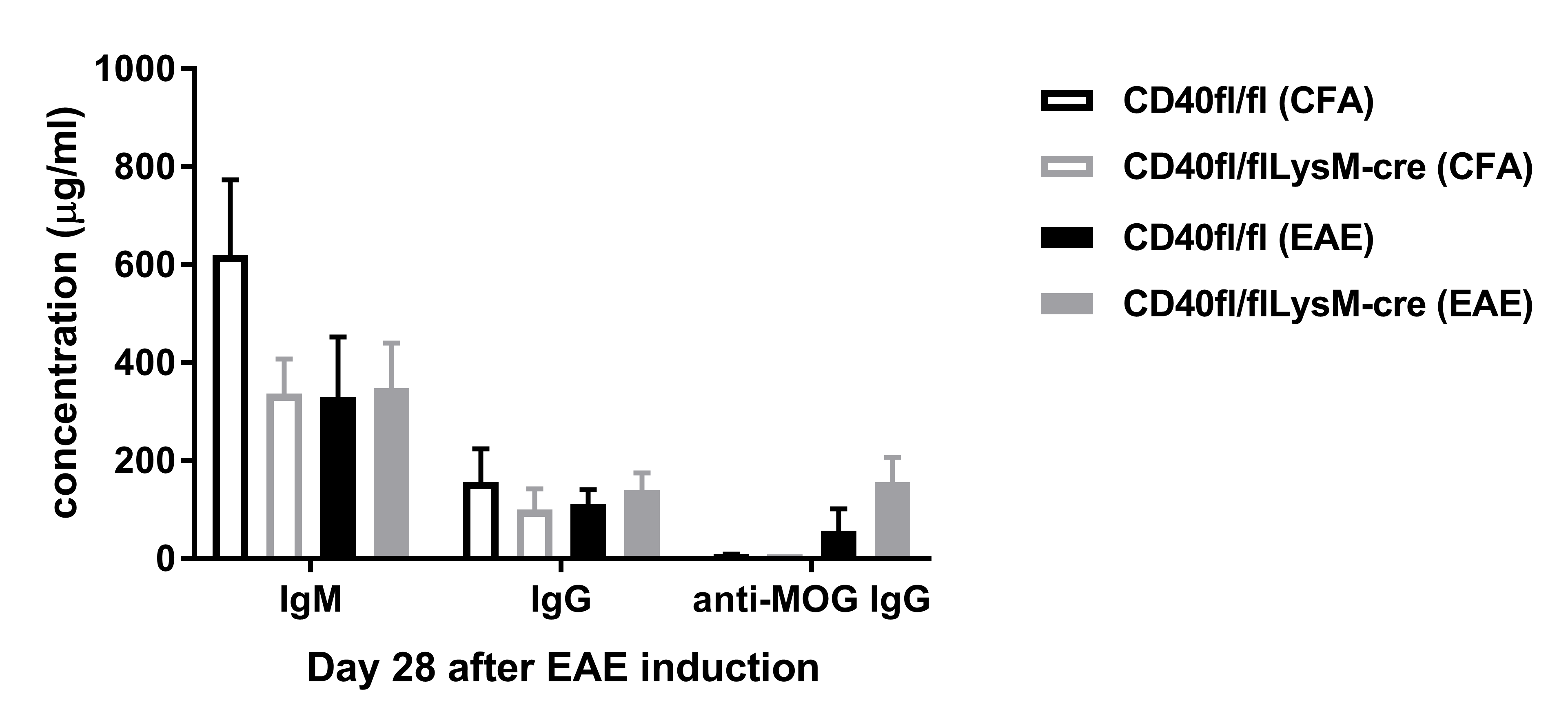

Supplement: Supplementary file 4 — Figure S3. Normal Ig isotype switching in CD40 flfl LysM cre mice [file PATH-247-471-s001.tif]

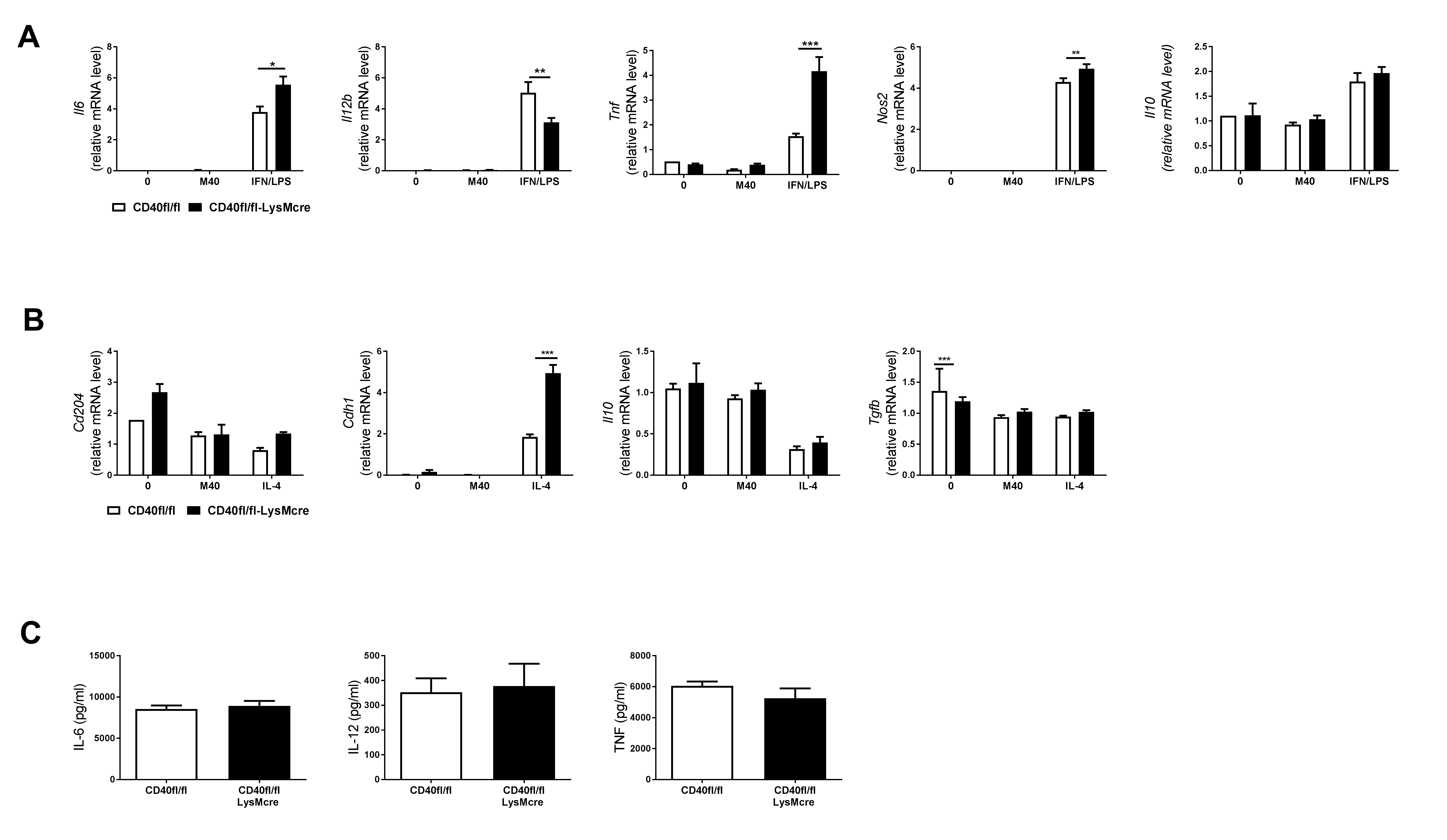

Supplement: Supplementary file 5 — Figure S4. CD40‐deficient macrophages have a diverse inflammatory pattern [file PATH-247-471-s002.tif]
