# Supplementary material for: Macrophage CD40 signaling drives experimental autoimmune encephalomyelitis
Source: J Pathol. 2019 Jan 30;247(4):471–80. doi: 10.1002/path.5205 (PMC6519352; doi:10.1002/path.5205)
Supplement: Supplementary file 6 — Table S1. Primer sequences [file PATH-247-471-s006.docx]

**Macrophage CD40 signaling drives experimental autoimmune encephalomyelitis**

Aarts SABM, *et al*. *J Pathol* 2018 (DOI: 10.1002/path.5205)

**Table S1.** Primer sequences

|  | **Forward** | **Reverse** |
| --- | --- | --- |
| ***Gapdh*** | 5’-CAACTCACTCAAGATTGTCAGCAA-3’ | 5’-TGGCAGTGATGGCATGGA-3’, |
| ***Rplp0*** | 5’-GGACCCGAGAAGACCTCCTT-3’ | 5’-GCACATCACTCAGAATTTCAATGG-3’ |
| ***Ifn-y*** | 5‘-GAGGAACTGGCAAAAGGATGG-3’ | 5‘-TGTTGCTGATGGCCTGATTG-3’ |
| ***Il17*** | 5'-TCCCTCTGTGATCTGGGAAG-3' | 5'-CTCGACCCTGAAAGTGAAGG-3' |
| ***Foxp3*** | 5'-CCCAGGAAAGACAGCAACCTT-3' | 5'-TTCTCACAACCAGGCCACTTG-3' |
| ***Mcp1*** | 5'-AGCACCAGCCAACTCTCACT-3' | 5' CGTTAACTGCATCTGGCTGA-3' |
| ***Tnf*** | 5'-CATCTTCTCAAAATTCGAGTGACAA-3' | 5’-TGGGAGTAGACAAGGTACAACCC-3’ |
| ***Il6*** | 5'-GCTACCAAACTGGATATAATCAGGAAA-3’ | 5'-CTTGTTATCTTTTAAGTTGTTCTTCATGTACTC-3’ |
| ***Il12b*** | 5’-GGTGCAAAGAAACATGGACTTG-3’ | 5’-CACATGTCACTGCCCGAGAGT-3’ |
| ***Nos2*** | 5’-GCAAACCCAAGGTCTACGTTCA-3’ | 5’-CCTCATTGGCCAGCTGCTT-3’ |
| ***Cd204*** | 5’-CAAATTGGCTTCCCTGGAGGT-3’ | 5’-CCACCAACCAGTCGAACTGTC-3’ |
| ***Cdh1*** | 5’-ATAATGACGCAGCTCAAGAATCTC-3’ | 5’-TTGTATTCGCCAATCTCTAAGTCC-3’ |
| ***Il10*** | 5’-TTTGAATTCCCTGGGTGAGAA-3’ | 5’-CTCCACTGCCTTGCTCTTATTTTC-3’ |
| ***Tgfb*** | 5’-GCCCTTCCTGCTCCTCATG-3’ | 5’-CCGCACACAGCAGTTCTTCTC-3’ |
